# Sarcopenia increases the risk for mortality in patients who undergo amputation for diabetic foot

**DOI:** 10.1186/s13047-018-0274-1

**Published:** 2018-06-19

**Authors:** You Keun Kim, Ho Seong Lee, Jae Jung Ryu, Hye In Lee, Sang Gyo Seo

**Affiliations:** 1 Department of Orthopedic Surgery, Asan Medical Center, University of Ulsan College of Medicine, Olympic-ro 43 gil 88, Seoul, 05505 Republic of Korea; 2 Diabetes Center, Asan Medical Center, University of Ulsan College of Medicine, Seoul, Republic of Korea

**Keywords:** Sarcopenia, Diabetic foot, Muscle, Amputation, Diabetes

## Abstract

**Background:**

Although there have been reports that diabetes affects the prevalence of sarcopenia, no studies have examined the relationship between sarcopenia and mortality in patients undergoing leg amputation. The purpose of this study is to determine whether sarcopenia affects the mortality rate of patients undergoing diabetic foot amputation.

**Methods:**

From among patients who underwent limb amputation for diabetes complications, this study included 167 patients who underwent abdominal CT within 1 year of amputation. We defined sarcopenia using sex-specific cut-off points for the L3 skeletal muscle index. The 5-year survival rate was analyzed. All patients were divided into two groups and compared according to the presence of sarcopenia. The mortality rate according to sarcopenia was assessed via the Kaplan-Meier method and log-rank test. Uni- and multivariate Cox regression analyses evaluated factors associated with survival rate.

**Results:**

Among the total of 167 patients, the overall 5-year mortality rate was 52.7%. Of the 112 patients with sarcopenia, the 5-year mortality rate was 60.7%. Of the 55 patients without sarcopenia, the 5-year mortality rate was 36.4%. Kaplan-Meier analysis showed a high mortality of the sarcopenia group in the univariate (*p* = 0.016) and multivariate (*p* = 0.047) analysis.

**Conclusions:**

Our study is the first to analyze the relationship between diabetic amputation and sarcopenia. Sarcopenia increases the risk of mortality in patients who undergo amputation for diabetic foot. Therefore, patients with diabetes should be careful to prevent sarcopenia with enough regular exercise as well as prevent diabetic foot disease.

## Background

The risk for developing diabetic foot ulcers in patients with diabetes over their lifetimes ranges from 19 to 34% [1–3]. Diabetic foot is associated with a very high mortality rate and is currently the main cause of nontraumatic lower limb amputation (LEA). [3] The mortality rate after LEA for diabetic foot was estimated at 13–40% within 1 year, 35–65% within 3 years, and 39–80% within 5 years, which is worse than that observed in malignancies [4]. Recently, Lavery et al. reported that mortality after diabetes-related amputation exceeds 70% at 5 years for all patients with diabetes. [5].

Previous studies have reported several factors that affect mortality after LEA [6–10]. Beyaz et al. reported that mortality could be predicted by the duration of insulin use, age, sex, and renal insufficiency after below-knee amputation [6]. Moreover, Costa et al. reported that old age, major amputation, and low hemoglobin levels are risk factors for death in patients with diabetic foot ulcers [7].

Sarcopenia is an aged related condition that involves multiple risk factors, and is associated with function decline, frailty, and other poor health outcomes [11, 12]. Sarcopenia is defined as the loss of skeletal muscle mass and strength with aging and contributes to both physical disability and mobility limitations [13]. In addition to aging, many chronic diseases are also associated with sarcopenia [12]. A few recent studies have shown that sarcopenia affects the outcomes including mortality of abdominal surgery, as well as solid organ cancer surgery [14–16]. Frailty that reduced homeostatic reserves has been studied with sarcopenia. When sarcopenia and frailty accompany, physical function impairment may eventually occur [17].

Among chronic diseases, diabetes is one of the significant contributors to the exacerbation of sarcopenia [17]. The Health, Aging, and Body Composition Study revealed that diabetes is associated with rapid loss of skeletal muscle strength and mass [18]. The prevalence of sarcopenia is as high as 15% in patients with type 2 diabetes [19]. Sarcopenia can cause a functional disability such as ambulation limitation after LEA, which is thought to be closely related to mortality.

However, the association between sarcopenia and mortality rate after LEA remains unclear. In addition, to the best of our knowledge, no study has investigated the effect of sarcopenia on postoperative outcomes including survival rate after LEA. If sarcopenia affects mortality after LEA, the presence of sarcopenia may be a predictor of the prognosis of LEA in patients with diabetic foot.

Therefore, the objective of this study was to evaluate the relationship between sarcopenia and mortality after LEA. The hypothesis was that sarcopenia affects the mortality of LEA.

## Methods

### Participants

This retrospective study was approved by the institutional review board of the Asan Medical Center. From 01/01/2005 to 31/12/2012, patients who underwent LEA due to diabetic complications and underwent abdominal computed tomography (CT) which is a valid measurement tool for sarcopenia were included. For evaluation about perioperative sarcopenia, patients who performed abdominal CT within 1 year before amputation surgery was selected. We excluded patients with solid organ malignancies detected via abdominal CT, owing to the possibility of the effects on mortality rate. As a result, only those patients who did not demonstrate any fatal abnormalities on CT were included. Patients with arteriosclerosis obliterans, Burger’s disease confirmed by angiography, trauma-related and skin cancer were excluded. Medical records were reviewed to collect basic demographic data such as sex, age, weight, height, and laboratory results including serum creatinine, HbA1c and fasting glucose level. The presence of relevant comorbidities including hypertension, cardiac disease, pulmonary disease, cerebrovascular disease and renal disease should also be reported. [20].

### Body composition and sarcopenia assessment

We defined sarcopenia by using sex-specific cut-off points for skeletal muscle index (SMI) at the level of the third lumbar vertebra (L3). L3 region is strongly correlated to whole body muscle distribution. As a result, the L3 slice allows for estimation of total muscle mass. [21] The L3 SMI was calculated as the total area of the L3 skeletal muscle area (cm^2^) divided by the height squared (m^2^). Abdominal CT images taken before LEA were retrieved for analysis. The CT-derived muscle mass at the level of L3 is closely correlated with bioimpedance on dual-energy X-ray absorptiometry at the level of L3 alone, whole-body bioimpedance [22]. The total area of muscle on a single axial slice at the level of L3, including the paraspinal, psoas, and abdominal wall musculature, has been employed for measurements in several previous studies [23]. The measurement of the skeletal muscle area was performed by using a PetaVision system (produced by Asan Medical Center, Seoul, Republic of Korea, ver 3.1.0.1152). Cutoffs of 52.4 cm^2^/m^2^ for men and 38.5 cm^2^/m^2^ for women were used on the basis of the findings from previous studies [24].

### Classification of LEA level

Amputation level was analyzed using medical records. Patients were divided into the major LEA group and minor LEA group according to the amputation level. The criteria for minor and major LEA were defined as at and below the ankle joint and above the ankle joint, respectively. For the purpose of this study, toe, ray, and transmetatarsal amputation was defined as minor LEA. Amputation below and above the knee was defined as major LEA.

### Outcome after LEA: Survival rate and functional score

The endpoint was survival rate. The overall 5-year mortality rate of patients who underwent LEA owing to diabetic complications was obtained. The date of death was confirmed based on the expiration date of medical insurance registration. All patients were divided into two groups and compared according to the presence or absence of sarcopenia as defined above.

### Statistical analysis

Statistical analysis was performed with SPSS 21.0 (SPSS Inc., Chicago, IL). A *p*-value < 0.05 was considered statistically significant. Differences in demographic parameters and FAAM scores between the two groups (sarcopenia group and non-sarcopenia group) were evaluated by using an independent two-sample t-test. The survival rate of all patients according to the presence of sarcopenia was assessed via the Kaplan-Meier method and log-rank test. The survival rates of the minor LEA and the major LEA group were also compared using the Kaplan-Meier method and log-rank test. Univariate and multivariate Cox regression analyses evaluated factors associated with survival rate along with the hazard ratios and 95% confidence intervals. Cox regression is method for investigating the effect of several variables upon the time a specified event takes to happen. In the context of an outcome such as death this is known as Cox regression for survival analysis.

## Results

### Demographics

The demographic data of the subjects included in the study are as follows. Of the 620 patients who underwent LEA within the study period, 216 who underwent abdominal CT were identified. We excluded 23 patients whose CT results were likely to affect survival, including those with malignant tumors. Of the remaining 193 patients, 167 were diagnosed with diabetes, 6 with arteriosclerosis obliterans, 11 with Burger’s disease, 2 with acute thromboembolism, 6 with trauma, and 1 with skin cancer. Finally, 167 patients were included in the study.

Of the 167 patients, 112 were male and 55 were female. The mean age of the patients at the time of LEA was 61.9 years (range, 29–80 years). Of the 167 patients, 112 had sarcopenia and the remaining 55 did not. Laboratory results including creatinine, HbA1c and fasting glucose level were confirmed. These laboratory result values were taken just before the amputation operation. The mean value of serum creatinine was 3.5 mg/dL, HbA1c was 7.6% and fasting glucose was 159.2 mg/dL. These laboratory measures showed no statistically significant difference between the sarcopenia and non-sarcopenia groups. And proportion of comorbidities including hypertension, cardiac disease, pulmonary disease, cerebrovascular disease and renal disease between the sarcopenia and non-sarcopenia groups showed no statistically different. Detailed proportions of comorbidity are listed in Table 1. Overall, 124 (74.3%) patients underwent minor LEA and 43 (25.7%) patients underwent major LEA. Detailed amputation levels are listed in Table 1.

**Table 1:** Demographics between patients with sarcopenia and without sarcopenia

Characteristics	Sarcopenia	Non-sarcopenia	*p*-value
Patients (number)	112 (67.1%)	55 (32.9%)	
Age (years)^a^	63.1 ± 11.6	60.5 ± 11.5	0.152
Gender, n			< 0.001
Male	90(53.9%)	28(16.8%)	
Female	22(13.2%)	27(16.2%)	
Weight (kg)^a^	57.2 ± 9.8	63.5 ± 9.3	< 0.001
Height (m)^a^	1.64 ± 0.08	1.62 ± 0.08	0.028
Body mass index, (kg/m^2^)^a^	21.1 ± 3.2	24.3 ± 3.1	< 0.001
Laboratory results			
Creatinine, mg/dL	3.6 ± 1.10	3.3 ± 0.71	0.157
HbA_1c_, % Fasting glucose level, mg/dL	7.6 ± 0.7 158.2 ± 23.3	7.6 ± 0.73 161.4 ± 25.8	0.851 0.421
Comorbidity			
Hypertension (yes/no)	36(32.1%) / 76(67.9%)	18(32.7%) / 37(67.3%)	0.981
Cardiac disease (yes/no)	27(24.1%) / 85(75.9%)		0.498
Pulmonary disease (yes/no)	10(8.9%) / 102(91.1%)	10(18.2%) / 45(81.8%)	0.514
Cerebrovascular disease (yes/no)	12(10.7%) / 100(89.3%)	7(12.7%) / 48(87.3%)	0.574
Renal disease (yes/no)	52(46.4%) / 60(53.6%)	4(7.3%) / 51(92.7%) 24(43.6%) / 31(56.4%)	0.582
Level of LEA			
Minor LEA (number)	78(69.6%)	46(83.6%)	
Toe	29	11	
Ray	27	25	
Transmetatarsal	22	10	
Major LEA (number)	34(30.4%)	9(16.4%)	
Below knee	29	8	
Above knee	5	1	

^a^Data are present as Mean ± SD [range], LEA: Lower extremity amputation

### Overall survival rate

**Fig. 1:**
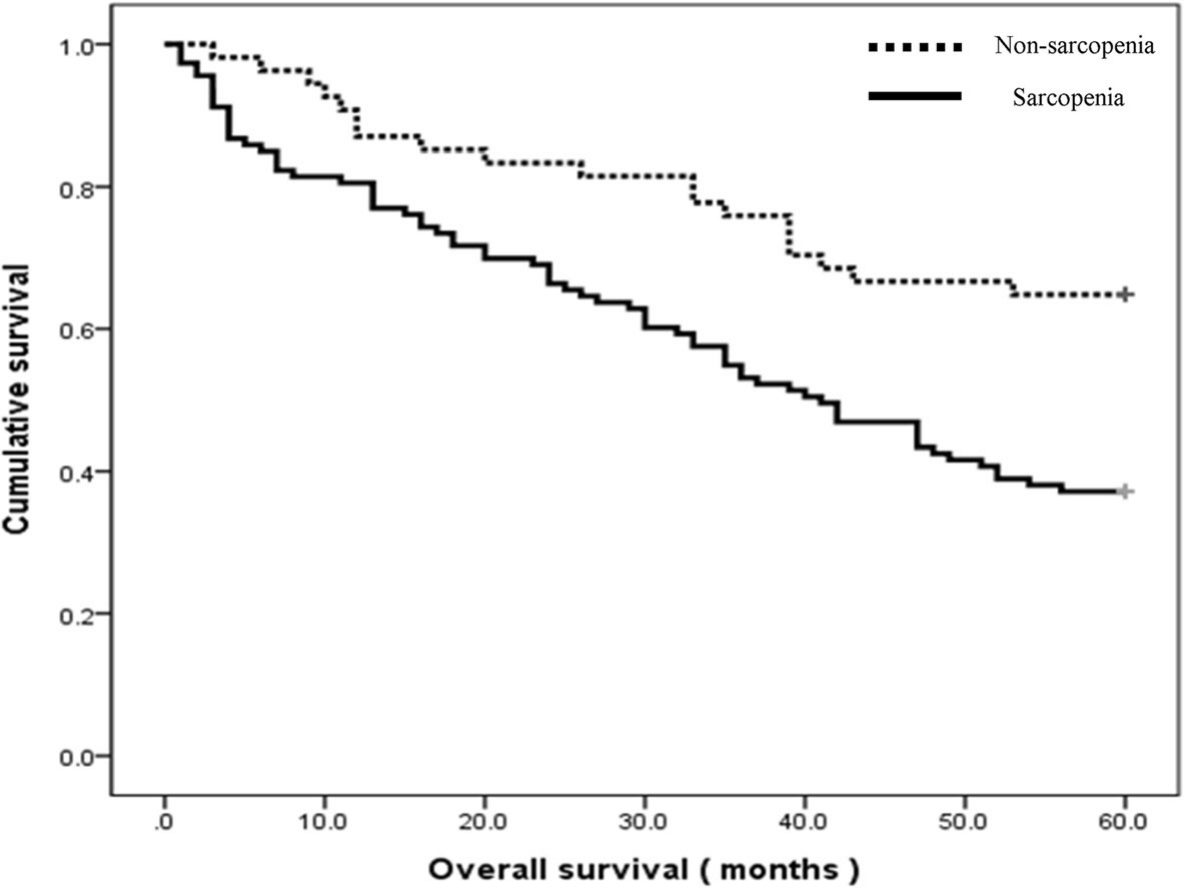
Kaplan-Meier survival graph for all subjects

Among the total of 167 patients, 88 patients died and 79 patients survived 5 years postoperatively. The overall 5-year mortality rate was 52.7%. In patients with sarcopenia, 5-year mortality rate was 60.7%. In patients without sarcopenia, 5-year mortality rate was 36.4% (Table 2). A Kaplan-Meier analysis showed a statistically significant difference between those with and without sarcopenia in the univariate (*p* = 0.016) and multivariate (*p* = 0.047) analysis (Fig. 1). Age and cardiac disease was a significant predictor of survival independent of sarcopenia in the univariate (*p* = 0.000) and multivariate (*p* = 0.003) analysis (Table 3).

**Table 2:** Mortality rate according to presence of sarcopenia and LEA level

	Total	5YMR	Sarcopenia			Non-sarcopenia			*p*-value
Patients (number)	167		112 (67.1%)			55 (32.9%)			
Survival			Expire	Survive	5YMR	Expire	Survive	5YMR	
Total	167	52.7%	68	44	60.7%	20	35	36.4%	0.006
Minor LEA	124	50.0%	45	33	57.7%	17	29	37.0%	0.007
Toe	40		19	10		2	9		
Ray	52		15	12		9	16		
Transmetatarsal	32		11	11		6	4		
Major LEA	43	60.5%	23	11	67.6%	3	6	33.3%	0.061
BKA.	37		21	8		3	5		
AKA.	6		2	3		0	1		

*5YMR*: 5-years mortality rate, *LEA*: lower extremity amputation, *BKA*: below knee amputation, *AKA*: above knee amputation

**Table 3:** Prognostic factors for mortality rate in patients undergoing an LEA for diabetic foot (univariate and multivariate analyses)

	Univariate		Multivariate	
Prognostic factor	*p*-value	HR (95% CI)	*p*-value	HR (95% CI)
Age	0.000	1.033 (1.014–1.052)	0.003	1.031 (1.010–1.053)
Gender	0.218	0.749 (0.473–1.186)	0.784	0.909 (0.458–1.803)
Body weight	0.378	1.009 (0.988–1.030)	0.030	1.029 (1.003–1.055)
Height	0.785	0.698 (0.052–9.302)	0.265	0.100 (0.002–5.760)
Sarcopenia	0.016	2.252 (1.161–4.369)	0.047	1.747 (1.008–3.027)
Creatinine	0.884	1.015(0.829–1.242)	0.388	1.091(0.895–1.330)
HbA_1C_	0.195	0.829(0.624–1.101)	0.285	0.857(0.646–1.137)
Fasting glucose	0.759	1.001(0.993–1.010)	0.701	1.002(0.993–1.011)
Hypertension	0.684	1.571(1.145–1.842)	0.517	0.842(0.065–1.145)
Cardiac disease	0.041	1.015(0.978–1.145)	0.034	1.175(1.012–1.248)
Pulmonary disease	0.248	2.154(1.784–2.426)	0.148	1.715(1.458–2.012)
Cerebrovascular disease	0.374	1.515(1.215–1.842)	0.314	1.126(0.865–1.458)
Renal disease	0.084	1.254(0.845–1.658)	0.145	1.548(1.256–1.842)

### Mortality rate according to amputation level

1. Minor LEA group (*n* = 124)
  In the minor LEA group, the 5-years mortality rate was 50.0%. In the minor LEA group with sarcopenia, 5-year mortality rate was 57.7%. And in the minor LEA group without sarcopenia, 5-year mortality rate was 37.0%. The 5-years mortality rate of patients undergoing minor amputation was significantly higher in those with sarcopenia (57.7%) compared to those without sarcopenia (37.0%) (*p* = 0.007; Fig. 2 and Table 2).

**Fig. 2:**
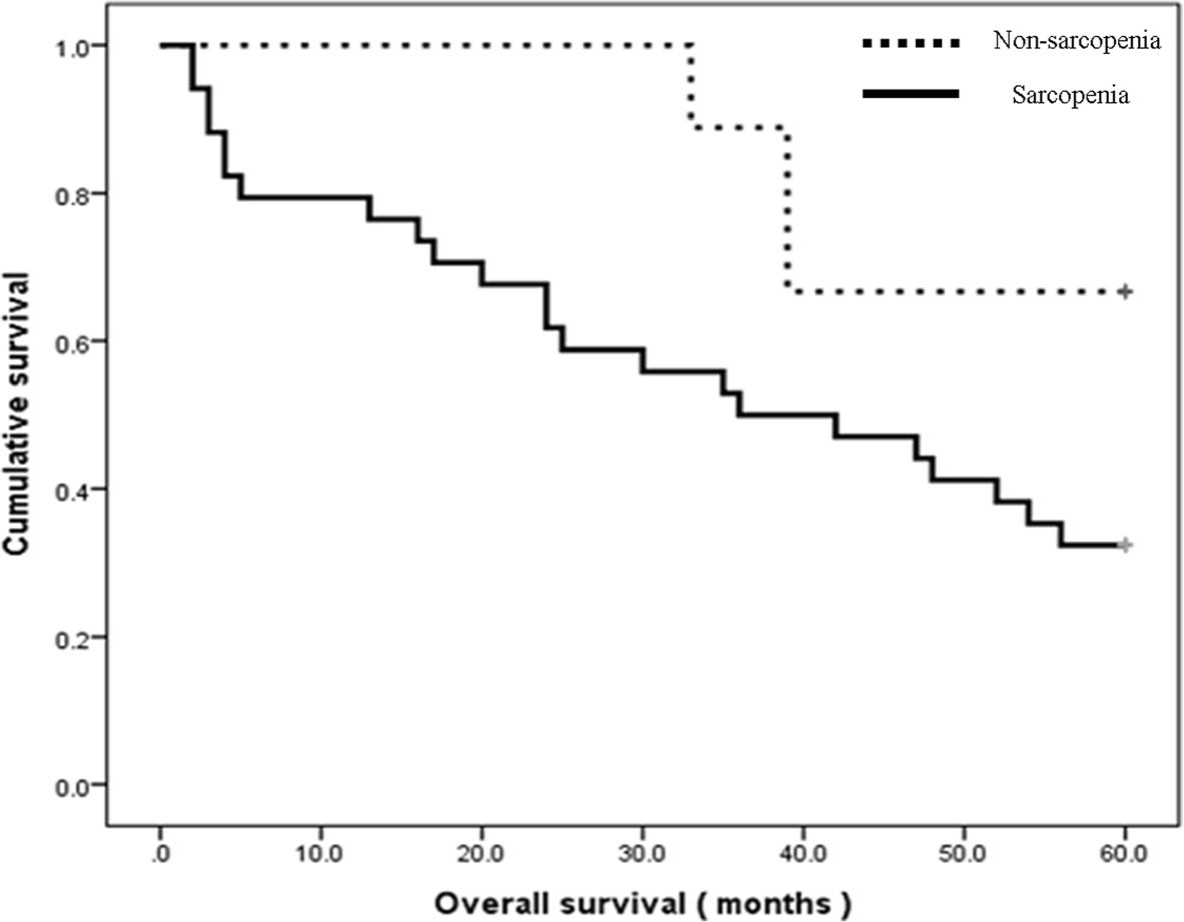
Kaplan-Meier survival graph for the minor amputation group
2. Major LEA group (*n* = 43)
  In the major LEA group, the 5-years mortality rate was 60.5%. In the major LEA group with sarcopenia, 5-year mortality rate was 67.6%. And in the major LEA group without sarcopenia, 5-year mortality rate was 33.3%. Although patients with sarcopenia who underwent major amputation had a higher mortality rate than patients without sarcopenia, this difference did not reach statistical significance with the numbers available (*p* = 0.061; Fig. 3 and Table 2).

**Fig. 3:**
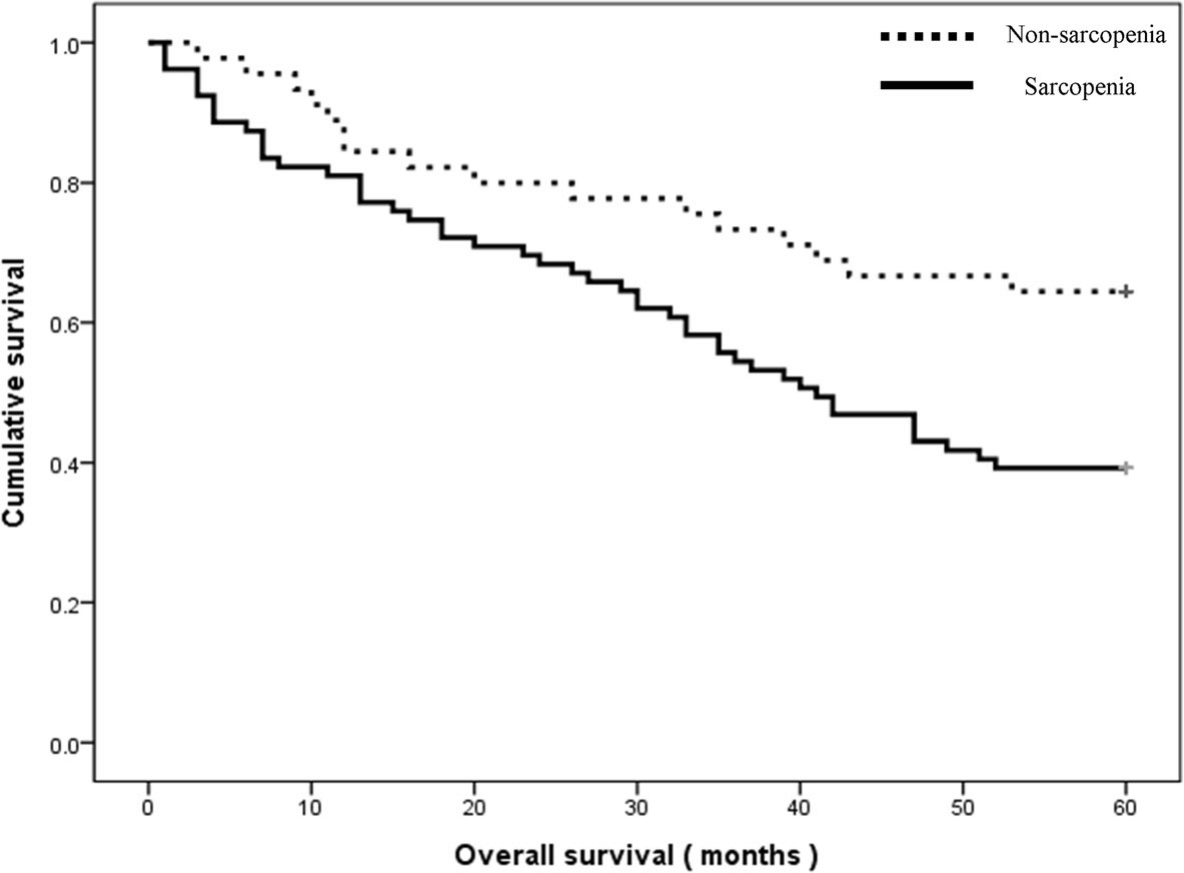
Kaplan-Meier survival graph for the major amputation group

## Discussion

To the best of our knowledge, this study is the first to analyze the relationship between survival after LEA and sarcopenia. The findings of this study showed that the mortality rate due to LEA in patients with sarcopenia was higher than that in patients without sarcopenia. These results indicate that preventing sarcopenia in diabetic patients is important to maintain high survival rates. For the same reason, the presence of sarcopenia can be a predictor of the outcome of LEA.

According to previous studies, the 5-year mortality rate after LEA was reported to be 39–80% [4, 25], and in our study, the 5-year mortality rate of all patients was 52.7%, similar to other studies. However, the 5-year mortality rate of patients with sarcopenia was only 60.7%, while the 5-year mortality rate of patients without sarcopenia was 36.4%. Even after adjusting for all other variables in the multivariate analysis, age, presence of cardiac disease and sarcopenia was an independent predictor for mortality in patients with LEA for diabetic foot. In previous studies [6–10, 26], the mortality rate was found to increase with higher amputation levels. Evans et al. [26] reported that the 2-year survival rate of forefoot/midfoot amputation group was 80%, while that of below knee amputation group was only 48%. However, in our study, the difference in mortality rate between patients who underwent minor amputation and those who underwent major amputation was 10.5%, whereas the difference in mortality rate according to the presence of sarcopenia was 24.3%. It means that mortality rates were significantly higher in sarcopenia patients regardless of the amputation level. And this suggests that the prevention or management of sarcopenia, regardless of amputation level, can be important to improve survival outcomes after LEA.

The known risk factors for survival after LEA are older age, male sex, long-term insulin use, and low hemoglobin level [6–10]. In our study, multivariate analysis was performed on factors that differed according to demographics such as age, sex, height, and weight, but only old age was found to have a significant effect. We also analyzed the relationship between laboratory results such as creatinine, HbA1c and fasting glucose level and survival rate, but no significant differences in survival rates were observed. There was no significant difference in preoperative creatinine level between sarcopenia and non-sarcopenia group, which is very valuable in that the presence of sarcopenia has a significant effect on survival, regardless of end-stage renal disease. It is not rejected by study by Wukich et al. [27] that end-stage renal disease affects survival. In our study, age, presence of cardiac disease and sarcopenia is an important independent predictor.

In a study of 414 type 2 diabetic patients aged 65 years and older, the risk for low muscle mass was 2–4 times higher in diabetic patients than in the control group [28]. It is known that comorbidities such as increased insulin resistance, higher cardiovascular risk factors, renal failure, peripheral neuropathy, and decreased muscle quality also influence prognosis [29]. However, this study showed that among patients with diabetes, the prognosis of patients with sarcopenia may be worse for the first time. Many patients with diabetic foot ulcer have relatively reduced activity, and their willingness and strength to walk especially after LEA becomes worse. On the basis of the findings of this study, we propose active rehabilitation and gait exercise training for patients with diabetic foot disease. In addition, our findings provide that efforts to improve muscle strength may increase the survival rate of patients with diabetic foot.

Although the survival rate is important, it is important to determine the treatment strategy of the patients, as well as the difference in functional activity among the surviving patients. Evans et al. [26] showed a difference in survival rates between below knee amputation group and forefoot/midfoot amputation group, but 64% were able to walk and the two groups were identical. This is contradictory to the general idea that functioning will drop when amputate at higher levels. This can be a hopeful message for patients who have undergone high level amputation. Wukich et al. [30] reported a significant improvement of SF-36 (Short Form 36) and FAAM score after major amputation compared with before amputation. However, the functional score was not included in this retrospective study.

This study has some limitations. First, there may have been selection bias because only those patients who underwent LEA and had CT data were included. Second, the amputation level was varied. However, we believe that our attempt to categorize amputation has the advantage of reflecting the overall progress of the patients. Third, in this study, only the muscle volume of L3 level was used as a diagnostic criterion, although the method of diagnosing sarcopenia is very diverse. Fourth, an analysis of the factors affecting the outcomes after LEA should include the presence of peripheral artery disease and evidence of infection. However, not all of the patients were not performed vascular study in this study and no available data about evidence of wound infection. Further study is therefore needed. Fifth, this study was retrospective in nature. Therefore, in addition to measuring the skeletal muscle index using CT, there was no investigation of muscle strength and performance, which is a recent diagnostic criterion for sarcopenia. Future prospective studies may be needed to investigate the relationship between muscle function and survival. Lastly, there was no preoperative and final follow up functional score such as FAAM score.

## Conclusions

Sarcopenia increases the risk of mortality in patients with LEA. Therefore, patients with diabetes should be careful to prevent sarcopenia with enough regular exercise as well as prevent diabetic foot disease.

## Abbreviations

CT: Computed tomography
FAAM: Foot and Ankle Ability Measure
LEA: Lower limb amputation
SMI: Skeletal muscle index

## References

[1] Frykberg RG, Zgonis T, Armstrong DG, Driver VR, Giurini JM, Kravitz SR, Landsman AS, Lavery LA, Moore JC, Schuberth JM (2006). Diabetic foot disorders. A clinical practice guideline (2006 revision). The J Foot and Ankle Surg : Official Pub of the American College of Foot and Ankle Surgeons.

[2] Wong E, Backholer K, Gearon E, Harding J, Freak-Poli R, Stevenson C, Peeters A (2013). Diabetes and risk of physical disability in adults: a systematic review and meta-analysis. Lancet Diabetes Endocrinol.

[3] Armstrong DG, Boulton AJM, Bus SA (2017). Diabetic foot ulcers and their recurrence. N Engl J Med.

[4] Singh N, Armstrong DG, Lipsky BA (2005). Preventing foot ulcers in patients with diabetes. Jama.

[5] Jupiter DC, Thorud JC, Buckley CJ, Shibuya N (2016). The impact of foot ulceration and amputation on mortality in diabetic patients. I: from ulceration to death, a systematic review. Int Wound J.

[6] Beyaz S, Guler UO, Bagir GS. Factors affecting lifespan following below-knee amputation in diabetic patients. Acta Orthop Traumatol Turc. 2017. 10.1016/j.aott.2017.07.001 PMC6197166 28865844

[7] Costa RHR, Cardoso NA, Procopio RJ, Navarro TP, Dardik A, de Loiola Cisneros L. Diabetic foot ulcer carries high amputation and mortality rates, particularly in the presence of advanced age, peripheral artery disease and anemia. Diabetes & metabolic syndrome. 2017; 10.1016/j.dsx.2017.04.008 28465149

[8] Nirantharakumar K, Saeed M, Wilson I, Marshall T, Coleman JJ (2013). In-hospital mortality and length of stay in patients with diabetes having foot disease. J Diabetes Complicat.

[9] Moulik PK, Mtonga R, Gill GV (2003). Amputation and mortality in new-onset diabetic foot ulcers stratified by etiology. Diabetes Care.

[10] Nather A, Bee CS, Huak CY, Chew JL, Lin CB, Neo S, Sim EY (2008). Epidemiology of diabetic foot problems and predictive factors for limb loss. J Diabetes Complicat.

[11] Liu P, Hao Q, Hai S, Wang H, Cao L, Dong B (2017). Sarcopenia as a predictor of all-cause mortality among community-dwelling older people: a systematic review and meta-analysis. Maturitas.

[12] Kalyani RR, Corriere M, Ferrucci L (2014). Age-related and disease-related muscle loss: the effect of diabetes, obesity, and other diseases. Lancet Diabetes Endocrinol.

[13] Cruz-Jentoft AJ, Baeyens JP, Bauer JM, Boirie Y, Cederholm T, Landi F, Martin FC, Michel JP, Rolland Y, Schneider SM (2010). Sarcopenia: European consensus on definition and diagnosis: report of the European working group on sarcopenia in older people. Age Ageing.

[14] Choi MH, Oh SN, Lee IK, Oh ST, Won DD. Sarcopenia is negatively associated with long-term outcomes in locally advanced rectal cancer. J Cachexia Sarcopenia Muscle. 2017. 10.1002/jcsm.12234 PMC5803619 28849630

[15] van Vugt JL, Braam HJ, van Oudheusden TR, Vestering A, Bollen TL, Wiezer MJ, de Hingh IH, van Ramshorst B, Boerma D (2015). Skeletal muscle depletion is associated with severe postoperative complications in patients undergoing Cytoreductive surgery with Hyperthermic intraperitoneal chemotherapy for peritoneal Carcinomatosis of colorectal Cancer. Ann Surg Oncol.

[16] Lieffers JR, Fassbender K, Winget M, Baracos VE, Bathe OF (2012). Sarcopenia is associated with postoperative infection and delayed recovery from colorectal cancer resection surgery. Br J Cancer.

[17] Bianchi L, Volpato S (2016). Muscle dysfunction in type 2 diabetes: a major threat to patient's mobility and independence. Acta Diabetol.

[18] Park SW, Goodpaster BH, Strotmeyer ES, Kuller LH, Broudeau R, Kammerer C, de Rekeneire N, Harris TB, Schwartz AV, Tylavsky FA (2007). Accelerated loss of skeletal muscle strength in older adults with type 2 diabetes: the health, aging, and body composition study. Diabetes Care.

[19] Kim TN, Park MS, Yang SJ, Yoo HJ, Kang HJ, Song W, Seo JA, Kim SG, Kim NH, Baik SH (2010). Prevalence and determinant factors of sarcopenia in patients with type 2 diabetes: the Korean Sarcopenic obesity study (KSOS). Diabetes Care.

[20] Jeffcoate WJ, Bus SA, Game FL, Hinchliffe RJ, Price PE, Schaper NC (2016). International working group on the diabetic F, the European wound management a: reporting standards of studies and papers on the prevention and management of foot ulcers in diabetes: required details and markers of good quality. Lancet Diabetes Endocrinol.

[21] Shen W, Punyanitya M, Wang Z, Gallagher D, St-Onge MP, Albu J, Heymsfield SB, Heshka S (2004). Total body skeletal muscle and adipose tissue volumes: estimation from a single abdominal cross-sectional image. J Appl Physiol.

[22] Mourtzakis M, Prado CM, Lieffers JR, Reiman T, McCargar LJ, Baracos VE (2008). A practical and precise approach to quantification of body composition in cancer patients using computed tomography images acquired during routine care. Appl Physiol Nutr Metab.

[23] Baumgartner RN, Koehler KM, Gallagher D, Romero L, Heymsfield SB, Ross RR, Garry PJ, Lindeman RD (1998). Epidemiology of sarcopenia among the elderly in New Mexico. Am J Epidemiol.

[24] Prado CM, Lieffers JR, McCargar LJ, Reiman T, Sawyer MB, Martin L, Baracos VE (2008). Prevalence and clinical implications of sarcopenic obesity in patients with solid tumours of the respiratory and gastrointestinal tracts: a population-based study. Lancet Oncol.

[25] Gazis A, Pound N, Macfarlane R, Treece K, Game F, Jeffcoate W (2004). Mortality in patients with diabetic neuropathic osteoarthropathy (Charcot foot). Diabetic medicine : a journal of the British Diabetic Association.

[26] Evans KK, Attinger CE, Al-Attar A, Salgado C, Chu CK, Mardini S, Neville R (2011). The importance of limb preservation in the diabetic population. J Diabetes Complicat.

[27] Wukich DK, Ahn J, Raspovic KM, Gottschalk FA, La Fontaine J, Lavery LA (2017). Comparison of Transtibial amputations in diabetic patients with and without end-stage renal disease. Foot Ankle Int.

[28] Kim KS, Park KS, Kim MJ, Kim SK, Cho YW, Park SW (2014). Type 2 diabetes is associated with low muscle mass in older adults. Geriatr Gerontol Int.

[29] Sinclair AJ, Abdelhafiz AH, Rodriguez-Manas L (2017). Frailty and sarcopenia - newly emerging and high impact complications of diabetes. J Diabetes Complicat.

[30] Wukich DK, Ahn J, Raspovic KM, La Fontaine J, Lavery LA (2017). Improved quality of life after Transtibial amputation in patients with diabetes-related foot complications. The Int J of Lower Extremity Wounds.

